# Structural barriers to work-family reconciliation in surgery: a gendered analysis of career disruption and care responsibilities

**DOI:** 10.1007/s13304-025-02489-3

**Published:** 2026-02-06

**Authors:** Valerie Isabel Nottberg, Lilly Klingelhöfer, Natasha Irene Schweitzer, Sarah Keller-Yamamura, Thilo Hackert, Isabel Molwitz, Jan Bardenhagen, Asmus Heumann

**Affiliations:** 1 Department of General, Visceral and Thoracic Surgery, University Medical Center Hamburg-Eppendorf, Martinistraße 52, 20246 Hamburg, Germany; 2 Department of Radiology, Charité Universitaetsmedizin Berlin, Corporate Member of Freie Universität Berlin, Humboldt-Universität zu Berlin, Berlin Institute of Health, Berlin, Germany; 3 Department of Diagnostic and Interventional Radiology and Nuclear Medicine, University Medical Center Hamburg-Eppendorf, Martinistraße 52, 20246 Hamburg, Germany

**Keywords:** Gender disparity, Surgery, Career development, Parental leave, Work-life balance, Childcare

## Abstract

Despite increasing numbers of women entering surgical training, gender disparities in surgical careers persist. Female surgeons remain underrepresented in leadership roles and face challenges related to family responsibilities. This study investigates gender-specific differences in surgical careers, family planning, caregiving responsibilities, and perceived institutional support in Germany. A nationwide cross-sectional survey was conducted among 432 board-certified surgeons and residents from various surgical specialties in Germany. A 68-item questionnaire assessed demographics, career stage, family planning, childcare responsibilities, work models, and perceived career barriers. Analyses included descriptive statistics, chi-square tests, t-tests, and regression models adjusted for age and institutional background. Female surgeons were significantly more likely to work part-time (OR 12.03, 95% CI 4.19–50.87), interrupt training due to parental leave, and experience prolonged specialization (OR 1.74, 95% CI 1.03–2.96). Nearly half (46.2%) postponed childbearing for career reasons (OR 4.72, 95% CI 2.34–10.23), and 82.5% reported negative career impacts from motherhood (OR 11.22, 95% CI 4.77–30.0). Women also reported greater domestic burdens, physical (OR 2.32, 95% CI 1.32–4.17) and emotional exhaustion (OR 2.61, 95% CI 1.39–5.20). Reassignment to non-surgical duties during pregnancy occurred in 46.4% (*n* = 44). Structural and cultural barriers constrain gender equity in surgery. Family responsibilities disproportionately affect female surgeons’ careers, contributing to slower advancement and increased burnout. Reforms such as flexible work models, accessible childcare, transparent promotion pathways, and mentorship are critical for fostering equal opportunity and retaining female talent for surgical leadership.

**Supplementary Information:**

The online version contains supplementary material available at 10.1007/s13304-025-02489-3.

## Introduction

### Structural challenges in a feminizing medical workforce

Since 2008, women have made up about half of all medical students in Germany [1]. This change reflects a combination of social changes that have increased women’s access to the medical profession. As the medical workforce becomes increasingly female, the rising workload presents significant challenges in balancing professional and family responsibilities, leaving many struggling to find a balance [2].

Surgery faces unique challenges in attracting, retaining, and promoting women into leadership positions [3]. At the same time, the healthcare system is under pressure owing to an aging population, which has significantly increased the demand for medical services. While various sectors have made progress in promoting workplace diversity and implementing family friendly policies, the medical field - especially surgical disciplines - has been slower to catch up [4].

Despite the increasing number of women entering surgery, they remain underrepresented in leadership roles. Annual reports from the German Medical Women’s Association have repeatedly highlighted the ongoing shortage of female leaders in university hospitals [5]. In surgical leadership positions, underrepresentation is especially pronounced: only 5% of surgical department chairs and 23% of senior attending surgeons in Germany are women. Furthermore, associate professorships in surgery are held by 16% female surgeons, compared to 84% male surgeons [5].

Women in academic medicine are 2.5 times more likely to report facing gender-based discrimination than their male counterparts. This troubling trend often stems from environments dominated by men, rigid hierarchies, and a general lack of awareness or accountability among leadership [6, 7].

### Family planning, care work, and risk of burnout

Family planning adds another layer of complexity to the careers of female surgeons. Unlike their male peers, women are less inclined to have children, and when they do, childbirth is often postponed to minimize disruptions during critical training periods [2, 8]. Nearly 80% of female surgical trainees indicate that pregnancy adversely affected their career progression. Moreover 30% would advise female medical students to pursue a surgical career altogether [9, 10].

Maternity leave is associated with prolonged training interruptions. Even after returning to work, women often find themselves shouldering the heavier burden of caregiving responsibilities well beyond the initial postpartum period. This imbalance can lead to chronic stress and limited career advancement [11]. These ongoing caregiving demands intensify the conflict between work and personal life, and are strongly associated with burnout, particularly among women [12]. Burnout and psychological distress were more often prevalent among female surgical trainees. Approximately 25% leave the profession before finishing their training [13], which discourages many young women from considering a surgical career [14, 15].

### Gender bias and attrition in surgical training

Cultural beliefs continue to play a significant role in maintaining these inequalities. Many studies indicate that surgery is still perceived as an unsuitable profession for women [16–18]. Even among women who do pursue a surgical career, dropout rates remain alarmingly high. Nearly one in four female surgical residents leaves training by the fourth year, compared to 17% of male residents [19]. Several factors contribute to this situation, such as poor work-life balance, lack of mentorship, inadequate support during pregnancy and parenthood, and an institutional culture that fails to foster the professional development of women [20, 21].

This study aimed to fill this critical gap by examining how family responsibilities influence the surgical careers of both women and men. Utilizing data from a nationwide survey, we investigate gender-specific trends in work arrangements, part-time employment, leadership paths, and caregiving duties. Our goal was to identify structural changes that promote greater gender equality in surgery.

## Methods

A nationwide, cross-sectional online survey was conducted between June and September 2024 among board-certified surgeons and surgical residents across Germany. We distributed invitations to 1754 surgical departments’ offices and individual surgeons across 48 institutions in Germany. The final sample comprised 432 participants (24.9%) from a range of surgical specialties, including visceral, traumatic, thoracic, cardiac, and plastic surgery. Further recruitment was conducted via professional networks. Respondents who were non-binary or did not specify their gender (*n* = 4) were excluded due to limited statistical representation. Item non-response was permitted; partially completed questionnaires were included. Consequently, n does not always equal 432 across outcomes (pairwise deletion).

The 68-item questionnaire, adapted from a previously validated instrument and expanded to address caregiving responsibilities, captures data on demographics, professional background, family obligations, and perceived career barriers [22, 23]. It included open and closed questions as well as assessments using a five-point Likert scale. Responses were collected anonymously using SurveyMonkey.

Statistical counsel was received. Statistical analyses were performed using IBM SPSS Statistics 29.0 (IBM Corp., Armonk, NY, USA) and R version 4.4.1 (2024-06-14) (R Foundation for Statistical Computing, Vienna, Austria). Categorical variables are presented as absolute and relative frequencies. Chi-square and independent-sample t-tests were used to assess group differences. Logistic and linear regression models were applied, adjusting for age and institution since there were significant differences in age and institutional background. Odds ratios (OR) with 95% confidence intervals (CI) were reported. Women were coded as 1 and men as 0 in regression models; thus, OR > 1 indicates higher odds in women . As this is an exploratory study, the p-values are descriptive. Ethical approval for the anonymous study was granted by local Ethics Committee in Hamburg (2025-101569-BO-ff). All participants indicated consent to participate in the study. All the analyses were conducted in compliance with the revised Declaration of Helsinki. This study followed the Strengthening the Reporting of Observational Studies in Epidemiology (STROBE) reporting guideline.

## Results

### Demographics and professional background

Of a total of 432 respondents, 73% (*n* = 315) were female and 27% (*n* = 117) male. The majority specialized in visceral surgery (46.1%), followed by general, trauma, and vascular surgery. Female surgeons were significantly less likely to be employed at university-affiliated medical centers compared to their male counterparts (53.5% vs. 88.8%; OR 0.11; 95% CI 0.05–0.21) and were underrepresented in senior roles, with only 2.9% attaining chief physician status versus 11.7% of men (OR 0.33; 95% CI 0.09–1.1).

Women were more likely to work part-time (25.5% vs. 5.4%; OR 12.03; 95% CI 4.19–50.87). They reported more frequent training interruptions due to parental leave often leading to extended residency durations (47.1% vs. 31.2%; OR 1.74; 95% CI 1.03–2.96). Key factors associated with prolonged training among women included part-time employment (27.6%), parental leave (42.5%), and job transitions (41.7% vs. 17.6%; OR 4.62; 95% CI 1.88–14.05). Details are reported in Table 1.

**Table 1:** Sociodemographic and professional characteristics of respondents by gender. (OR = Odds ratio; CI = Confidence interval, Ref. = reference, NA = not applicable, * indicates *p* < 0.05 (two-sided)*)*

	Female *N* / %	Male *N* / %	Total *N* / %	*p* < 0.05*	OR 95% CI
	315 / 73%	116 / 27%	432 / 100%		
**Year of birth**					
1950–1969	14 / 6.2%	11 / 12.5%	26 / 8.2%		NA
1970–1979	36 / 15.9%	19 / 21.6%	56 / 17.6%		NA
1980–1989	106 / 46.7%	40 / 45.5%	147 / 46.1%		NA
1990–1999	71 / 31.3%	18 / 20.5%	89 / 27.9%		NA
After 2000	0 / 0%	0 / 0%	1 / 0.3%		NA
**Are you currently employed at a university hospital?**					
Yes	167 / 53.5%	103 / 88.8%	274 / 63.3%	*	**0.11 (0.05–0.21)**
No	144 / 46.2%	13 / 11.2%	158 / 36.5%		Ref
**Current professional position** *The reference category consists of respondents who did not choose the respective option.*					
Resident physician	102 / 37.4%	26 / 23.4%	128 / 33%		1.04 (0.45–2.5)
Board-certified specialist	55 / 20.1%	10 / 9.0%	66 / 17%	*	**2.17 (1.06–4.87)**
Senior physician / Consultant	90 / 33.0%	41 / 36.9%	133 / 34.3%		1.17 (0.66–2.07)
Senior consultant / Deputy head of department	16 / 5.9%	20 / 18%	36 / 9.3%	*	**0.37 (0.16–0.88)**
Head of department / Chief physician	8 / 2.9%	13 / 11.7%	21 / 5.4%		0.33 (0.09–1.1)
**What is your surgical subspecialty? (multiple answers possible)** *The reference category consists of respondents who did not choose the respective option.*					
General surgery	63 / 22.8%	39 / 35.8%	102 / 26.3%		0.67 (0.37–1.22)
Vascular surgery	42 / 15.2%	18 / 15.6%	60 / 15.5%		0.98 (0.48–1.9)
Cardiac surgery	13 / 4.7%	14 / 12.8%	27 / 7%	*	**0.32 (0.12–0.82)**
Trauma surgery	60 / 21.7%	23 / 21.1%	85 / 21.9%		1.05 (0.58–1.96)
Visceral surgery	123 / 44.6%	56 / 51.4%	179 / 46.1%		0.81 (0.48–1.34)
Thoracic surgery	19 / 6.9%	15 / 13.8%	34 / 8.8%		0.81 (0.35–2.02)
Plastic surgery	13 / 4.7%	1 / 0.9%	15 / 3.9%		3.2 (0.53–61.8)
**What is your academic qualification or title** *The reference category consists of respondents who did not choose the respective option.*					
Research associate / Academic staff	33 / 12.4%	10 / 9.0%	43 / 11.3%		1.12 (0.49–2.69)
Doctorate	99 / 37.2%	36 / 32.4%	138 / 36.2%	*	**2.64 (1.44–4.92)**
Assistant professor	13 / 4.9%	19 / 17.1%	32 / 8.4%		0.48 (0.2–1.15)
Associate professorship	7 / 2.6%	16 / 14.4%	23 / 6.0%		0.38 (0.12–1.14)
Full university professorship	3 / 1.1%	14 / 12.6%	17 / 4.5%	*	**0.2 (0.04–0.73)**
No answer	111 / 41.7%	16 / 14.4%	128 / 33.6%		NA
**Has your specialist training taken longer than expected or is it expected to take longer?**					
Yes	129 / 47.1%	34 / 31.2%	165 / 42.6%	*	**1.74 (1.03–2.96)**
No	145 / 52.9%	75 / 63.8%	222 / 57.4%	*	Ref
**What were or are the reasons for the extension** *The reference category consists of respondents who did not choose the respective option.*					
Job change	53 / 41.7%	6 / 17.6%	59 / 36.2%	*	**4.62 (1.88–14.05)**
Part-time work	35 / 27.6%	0 / 0%	36 / 22.1%		NA
Parental leave	54 / 42.5%	7 / 20.6%	63 / 38.7%	*	**3.04 (1.32–8.26)**
Unavailability of rotation placements	14 / 11%	6 / 17.6%	20 / 12.3%		1.18 (0.39–3.21)
Illness	3 / 2.4%	1 / 2.9%	4 / 2.4%		1.65 (0.2–34.33)
Research	25 / 19.7%	20 / 58.8%	45 / 27.6%	*	**0.34 (0.17–0.7)**
Stay abroad	14 / 11%	10 / 29.4%	28 / 17.2%	*	0.35 (0.13–0.95)
Other	37 / 29.1%	11 / 32.4%	48 / 29.4%		NA
**Do you work full-time or part-time?**					
Full-time	202 / 74.5%	105 / 94.6%	308 / 79.8%	*	Ref
Part-time	69 / 25.5%	6 / 5.4%	78 / 20.2%	*	**12.03 (4.19–50.87**)

### Family planning, partnerships, and care responsibilities

Most respondents were either married or had committed partnerships (*n* = 266, 83.4%). A significantly higher proportion of male surgeons were married compared to their female colleagues (77.3% vs. 47.6%; OR 0.26; 95% CI 0.14–0.48). Among female surgeons, 97.8% (*n* = 177) reported that their partners were employed, but only 16.4% (*n* = 29) of female surgeons’ partners worked part-time (OR 0.19; 95% CI 0.10–0.36). In contrast, 85% (*n* = 68) of male surgeons had employed partners, who worked part-time in most cases (52.3%; *n* = 36). Details are reported in Table 2.

**Table 2:** Relationship status, partner’s employment, and working hours by gender. (OR = Odds ratio; CI = Confidence interval, Ref. = reference, NA = not applicable, * indicates *p* < 0.05 (two-sided))

	Female *N* / %	Male *N* / %	Total *N* / %	*p* < 0.05*	OR 95% CI
**What is your marital status?** *The reference category consists of respondents who did not choose the respective option*					
Married	108 / 47.6%	68 / 77.3%	179 / 56.1%	*	**0.26 (0.14–0.48)**
In a committed relationship	73 / 32.5%	13 / 14.8%	87 / 27.3%	*	**2.31 (1.2–4.71)**
Single / divorced / widowed	3 / 1.3%	1 / 1.1%	5 / 1.6%	*	**4.06 (1.55–13.97)**
**Is your partner currently employed?** *The reference category consists of respondents who did not choose the respective option*					
Yes	177 / 97.8%	68 / 85%	248 / 93.6%	*	**11.36 (3.35–52.28)**
No	4 / 2.2%	12 / 15%	16 / 6%		Ref
**What is your partner’s current working time?** *The reference category consists of respondents who did not choose the respective option*					
Full-time	148 / 83.6%	33 / 47.8%	184 / 73.6%		Ref
Part-time	29 / 16.4%	36 / 52.3%	66 / 26.4%	*	**0.19 (0.1–0.36)**
**Is your partner also working as a physician?** *The reference category consists of respondents who did not choose the respective option*					
Yes	52 / 29.5%	31 / 44.9%	84 / 33.7%	*	**0.51 (0.28–0.92)**
No	124 / 70.5%	37 / 53.6%	163 / 65.5%		Ref
**Do you work full-time or part-time?**					
Full-time	202 / 74.5%	105 / 94.6%	308 / 79.8%		Ref
Part-time	69 / 25.5%	6 / 5.4%	78 / 20.2%	*	**12.03 (4.19–50.87**)

**Fig. 1:**
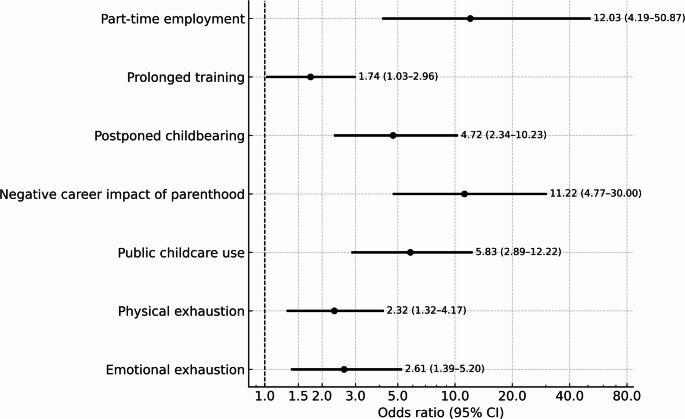
Overview of key gender-associated effects: log-scaled adjusted odds ratios (OR) with 95% confidence intervals for women vs. men across (reference = men) in seven outcomes (employment, training, family planning, caregiving, and well-being). The dashed vertical line marks OR = 1; OR > 1 indicates higher odds in women

Female surgeons were significantly more likely to postpone family planning for career-related reasons than their male counterparts (46.2% vs. 14.7%; OR 4.72; 95% CI 2.34–10.23). Compared to male surgeons, they tended to have children later during residency without holding leadership positions at the time of childbirth (OR 2.46; 95% CI 1.08–6.02). Women were also more frequently childless (53.7% vs. 25.6%) and had fewer children than men (1.7 ± 0.8 vs. 2.3 ± 1.0; β = − 0.55). Public childcare services were used significantly more often by female surgeons (68.8% vs. 28.6%; OR 5.83; 95% CI 2.89–12.22), whereas male surgeons predominantly relied on their partners for childrearing (54%; OR 0.03; 95% CI 0.01–0.09) (Table 3). Female surgeons took on most organizational responsibilities related to their children (71.3% vs. 6.3%; OR 37.61; 95% CI 13.58–135.53), and were more frequently to stay at home when a child was sick (26.1% vs. 4.8%; OR 6.8; 95% CI 2.21–29.83). Main findings are shown in Fig. 1.

**Table 3:** Parenthood, childcare responsibilities, and career interruptions by gender. (OR = Odds ratio; CI = Confidence interval, Ref. = reference, NA = not applicable, * indicates *p* < 0.05 (two-sided)*)*

	Female *N* / %	Male *N* / %	Total *N* / %	*p* < 0.05*	OR 95% CI
**Have you postponed having children for career reasons?**					
Yes	96 / 46.2%	11 / 14.7%	108 / 37.8%	*	**4.72 (2.34–10.23)**
No	99 / 47.6%	60 / 80%	160 / 55.9%	*	Ref
No answer	13 / 6.3%	4 / 5.3%	18 / 6.3%		
**Do you have children?**					
Yes	96 / 44.9%	64 / 74.4%	162 / 53.5%	*	**0.26 (0.12–0.5)**
No	115 / 53.7%	22 / 25.6%	137 / 45.2%	*	Ref
**How are/were your children mainly cared for during the day?** *The reference category consists of respondents who did not choose the respective option.*					
By myself	7 / 7.5%	0 / 0%	7 / 7.5%		NA
By my partner	3 / 3.2%	34 / 54%	37 / 23.4%	*	**0.03 (0.01–0.09)**
By other family members	2 / 2.2%	1 / 1.6%	3 / 1.9%		1.32 (0.11–31.14)
By a nanny / au-pair / friend	12 / 12.9%	5 / 7.9%	17 / 10.8%		1.93 (0.66–6.47)
By a public childcare institution	64 / 68.8%	18 / 28.6%	84 / 53.2%	*	**5.83 (2.89–12.22)**
**Have you or your partner ever interrupted work due to childcare (e.g. maternity/paternity leave)?** *The reference category consists of respondents who did not choose the respective option.*					
I interrupted my own work.	89 / 94.7%	28 / 44.4%	119 / 74.8%		**2.3 (0.09–59.45)**
My partner interrupted their work	39 / 41.5%	43 / 68.3%	83 / 52.2%	*	**0.2 (0.11–0.36)**
My partner stopped working entirely to care for the child	1 / 1.1%	5 / 7.9%	6 / 3.8%	*	**0.06 (0–0.42)**
Neither of us interrupted work	2 / 2.1%	6 / 9.5%	8 / 5%	*	**0.17 (0.02–0.8)**

Regarding unpaid domestic labor, female surgeons reported spending an average of 18.4 ± 17.9 h per week on household tasks and childcare, substantially more than their male counterparts, who reported 13.7 ± 13.2 h (β = 5.49). Women estimated that their partners contributed approximately 14.4 ± 17.3 h weekly. In contrast, male surgeons reported that their partners performed 31.6 ± 33.3 h of domestic work per week.

In the event of a child’s illness, 26.1% (*n* = 24) of female surgeons reported primary caregiving responsibilities, compared to only 4.8% (*n* = 3) of male surgeons (OR 6.8; 95% CI 2.21–29.83). Among male surgeons, the responsibility was most often delegated to their partners (47.6%, *n* = 30), whereas this was the case for only 12% (*n* = 11) of female surgeons’ partners (OR 0.14; 95% CI 0.06–0.31). Shared caregiving by both parents was reported by 41.3% (*n* = 26) of male surgeons and 57.6% (*n* = 53) of female surgeons.

Female surgeons expressed a willingness to increase their working hours if reliable childcare was available (34% vs. 7.9%; OR 5.76; 95% CI 2.16–18.38). Career interruptions due to childbearing were longer among women, averaging 19.3 ± 13.1 months compared to 2.7 ± 2.7 months for men.

### Dual-physician households and gendered career impact

Eighty-three respondents were in dual-physician couples, of whom 52 female surgeons (26.8%) and 31 male surgeons (26.7%) were married or in a partnership with another doctor. Among female surgeons partnered with male physicians, 86.5% (*n* = 45) reported their partners worked full-time, while 13.5% (*n* = 7) worked part-time. Career-related concerns led 28.8% (*n* = 15) to forgo and 45.8% (*n* = 23) to postpone having children. Of those with children (62.5% *n* = 32), most relied on public childcare (65.4%, *n* = 34), with minimal involvement from partners (3.8%, *n* = 2). The majority (76.9%, *n* = 40) of female surgeons managed family logistics, and 44.2% (*n* = 23) stayed home when children were sick - compared to only one male partner (3.2%) (*p* < 0.01). Nearly all women (92.3%, *n* = 48) had taken career breaks for childcare, versus 32.6% (*n* = 17) of their male partners (*p* < 0.01). Additionally, 46.1% (*n* = 24) said they would increase work hours if adequate childcare was available. Only 9.0% of male surgeons expressed willingness to increase work hours with better childcare.

In contrast, among male surgeons with female physician partners (*n* = 31), 22.6% (*n* = 7) had forgone and 22.6% (*n* = 7) had postponed childbearing, and 77.4% (*n* = 24) had children. In most cases, the female partner provided primary care (54.8%, *n* = 17) and handled family logistics (80.6%, *n* = 25). While parental leave was taken by both partners in most cases, women were more often the ones to stay home when children were ill (26.9%, *n* = 14 vs. 9.7%, *n* = 3).

### Perceived career obstacles and emotional well-being

There were no significant differences between the female and male surgeons regarding their expectations of professional training quality. Better work-life balance was a more important factor for female surgeons than for male surgeons (30.8%, *n* = 66 vs. 7.3%, *n* = 14; OR 2.27; 95% CI 1.2–4.51). Predictable working hours were also rated as more important by women (OR 1.89; 95% CI 1.11–3.11). When asked whether these expectations were being met, approximately 60% of both female and male surgeons perceived their profession as poorly compatible with family life (OR 0.96; 95% CI 0.56–1.66), and more than half felt that predictable working hours were rarely achievable (OR 1.14; 95% CI 0.66–2.03).

**Fig. 2:**
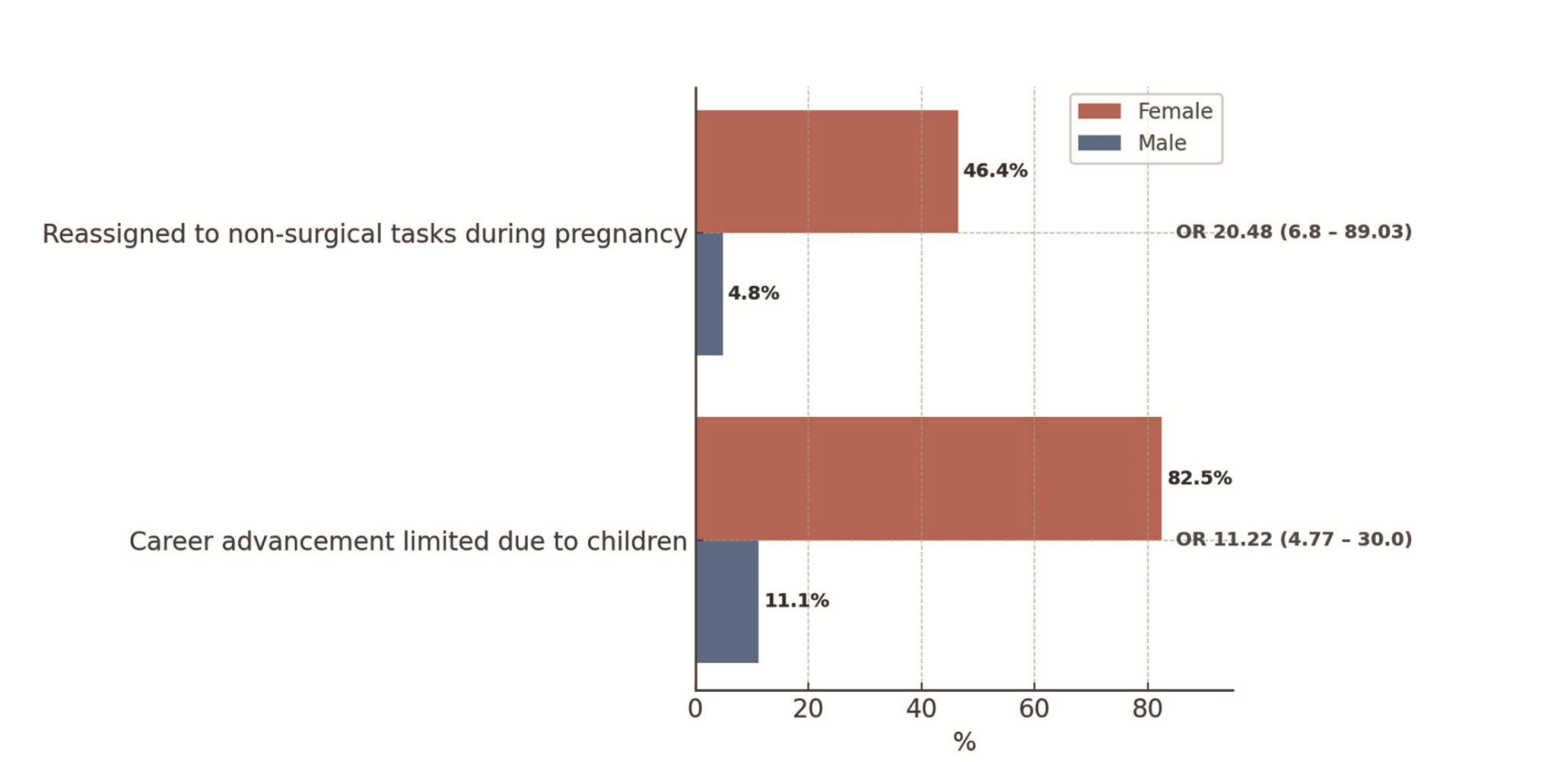
Perceived impact of personal pregnancy (in females), partner’s pregnancy (in males) and parenthood on surgical career progression by gender showing answers with “yes”

Female surgeons reported more career disadvantages related to pregnancy and parenthood than male surgeons. Nearly half had been reassigned to non-surgical duties during their pregnancy (46.4%, *n* = 44). Additionally, 82.5% (*n* = 52) of female respondents stated that having children had negatively impacted or hindered their career progression, compared to only 11.1% of male colleagues (OR 11.22; 95% CI 4.77–30) (Fig. 2).

**Fig. 3:**
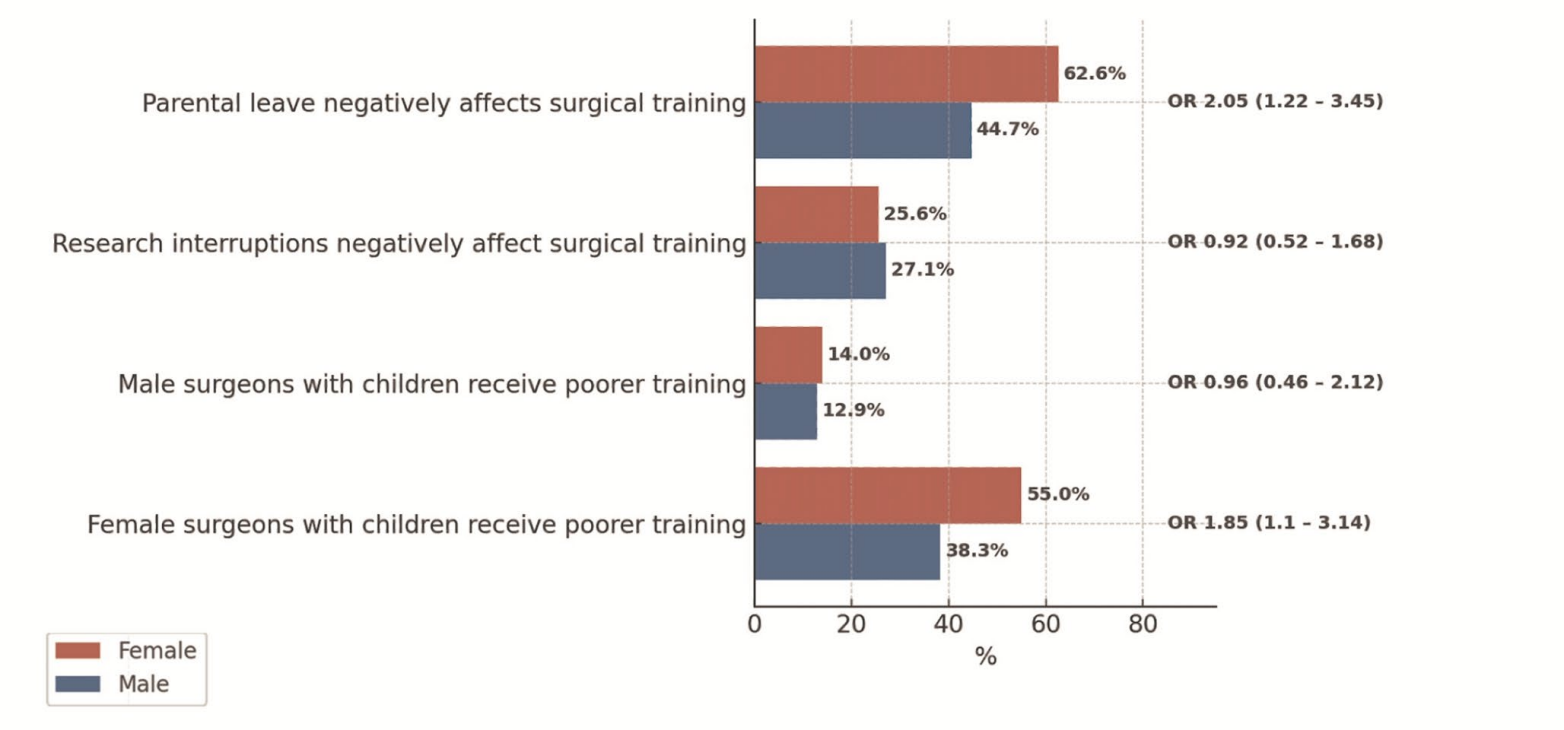
Gender-specific distribution of career interruptions due to parental leave, surgical education and resulting consequences

Most female respondents (62.6%) strongly agreed that child-related absences impaired surgical training, compared to 44.7% of men (OR 2.05; 95% CI 1.22–3.45). Moreover, 55.0% of women believed that mothers in surgery receive inferior training opportunities, while only 38.3% of men shared this view (OR 1.85; 95% CI 1.1–3.14) (Fig. 3).

Women were more likely than their male counterparts to report both physical (OR 2.32; 95% CI 1.32–4.17) and emotional exhaustion (OR 2.61; 95% CI 1.39–5.20). They were also less likely to perceive their work as meaningful, with only 22.4% affirming this sentiment compared to 35.8% of men (OR 0.56; 95% CI 0.32–1.00). Notably, more than half of all participants, regardless of gender, reported continuing to work while ill.

### Social support and leadership preferences

Both genders reported comparable levels of peer support; however, women felt less supported by their partners (50.2% vs. 75.3%, OR 0.31; 95% CI 0.17–0.56), families (42.1% vs. 65.4%, OR 0.38; 95% CI 0.21–0.65), and supervisors (15% vs. 25%, OR 0.52; 95% CI 0.28–1.04).

**Fig. 4:**
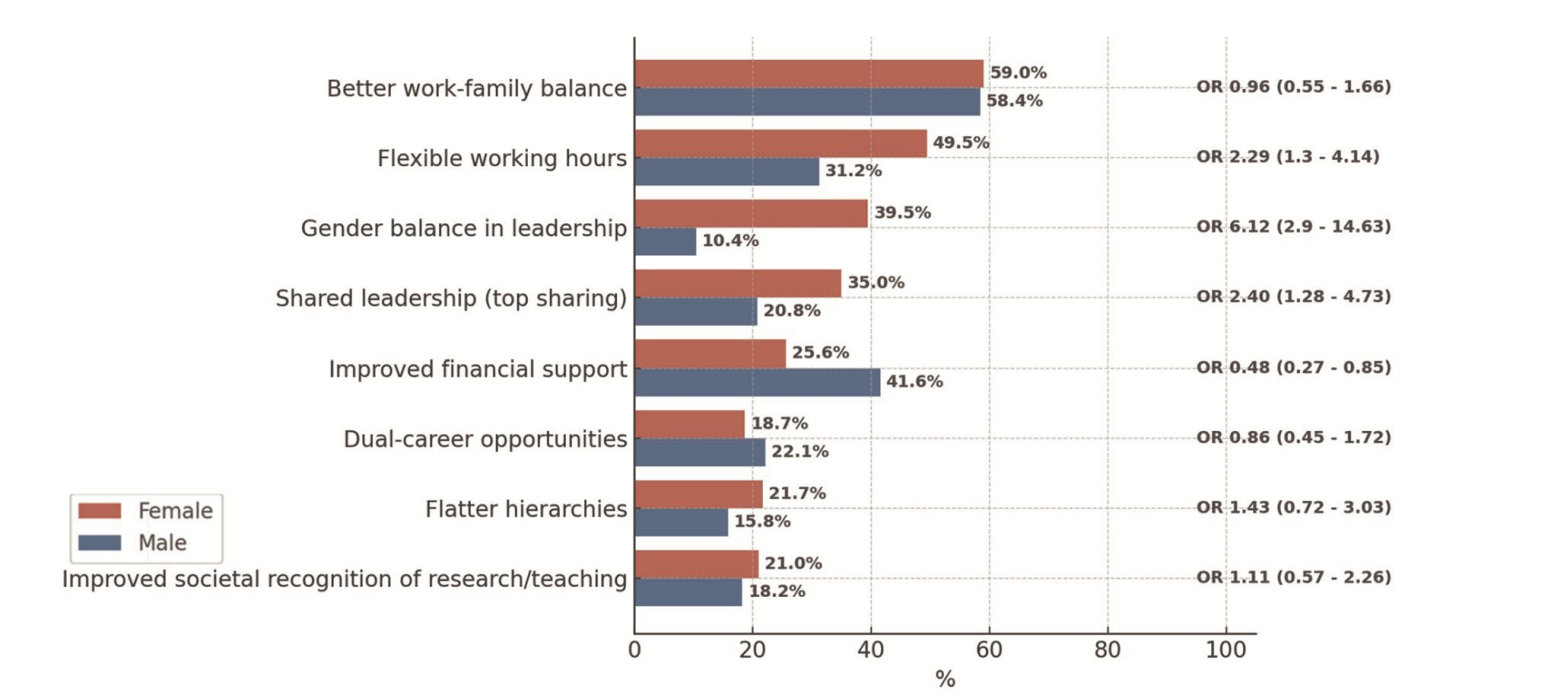
Perceived effectiveness of measures to enhance the attractiveness of leadership positions in academic medicine. Proportion of respondents who rated selected measures as “very helpful,” shown by gender

When evaluating strategies to enhance the appeal of leadership roles, women expressed a stronger preference for flatter hierarchies, job-sharing models (“top sharing,” OR 2.40; 95% CI 1.28–4.73), gender-balanced executive teams (OR 6.12; 95% CI 2.90–14.63), and flexible working arrangements (OR 2.29; 95% CI 1.30–4.14). By contrast, men placed greater emphasis on improving financial compensation. Both groups saw the importance of balancing career and family lives (Fig. 4).

## Discussion

This study evaluated how family responsibilities influence the surgical careers of both women and men utilizing data from a nationwide survey and investigated gender-specific trends in work arrangements, part-time employment, leadership paths, and caregiving duties. In addition, we provide actionable aims based on informed strategies from this survey.

### Gendered career realities

This study presented a large-scale investigation comparing the professional realities of male and female surgeons in Germany. With most female participants (72.6%), it offered unique insight into gender-specific barriers that extend beyond general challenges in surgical careers. By including a group of male surgeons for comparison, the study enhances its analytical depth, by focusing on structural inequalities rather than attributing disparities to individual choices. The cohort represents a wide range of demographics, from early career to senior surgeons across various institutional settings.

### Structural barriers and the “Second Shift”

Our research highlights clear gender differences in family planning, sharing of household duties, and perceptions of career growth. Female surgeons were more likely to postpone or forgo having children due to professional obligations, resulting in a higher incidence of childlessness. Even among those who are parents, women took on most of the responsibilities for household tasks, managing children’s schedules, and caring for sick children. In dual-physician households, female partners handled approximately 80% of family obligations, despite both partners working full-time in demanding roles.

This unequal division of labor illustrates the ongoing “second shift” that women experience - where caregiving tasks add to their already busy workdays. Male surgeons often have the advantage of partners working part-time and taking on a large share of their domestic responsibilities. This imbalance translated directly into professional disparities: women reported having to interrupt their training more often and their residency periods were longer. Many perceived their career advancement to be hindered by parenthood, an assessment caused by institutional reassignment to non-surgical tasks during pregnancy.

Our data also indicate a concerning gender gap in well-being: female surgeons reported higher levels of physical and emotional exhaustion. This reflects the combined strain of juggling dual roles and the limited structural support. Previous studies have shown that female physicians with children face a higher risk of burnout and depressive symptoms [11]. In our cohort, female surgeons were more than twice as likely as males to report frequent exhaustion, a finding that aligns with U.S. data, where 43.3% of female surgeons compared to 39% of male surgeons experienced burnout [24]. Although personal resilience strategies may offer some benefit, they are insufficient in the absence of structural changes. Institutions must address the unequal distribution of workload and create targeted support systems to reduce burnout and improve the long-term retention of female surgical talent [25].

These patterns are reinforced by persistent patriarchal norms and rigid institutional structures within hospital organizations, which continue to hinder women’s professional development [26]. As a result, even female surgeons themselves often believe that maternity leave and caregiving negatively affect the quality of surgical training. Importantly, the underrepresentation of women in leadership roles is not due to a deficiency in their ambition or capability. Even though they often achieve better academic results, including higher exam scores and more doctoral degrees than their male counterparts, women still face obstacles in climbing the clinical or academic ladders [27].

Across Europe, the Nordic welfare states are often considered frontrunners; yet even there, surgical training and careers show clear gender imbalances [28]. Norwegian pipeline data and Swedish evidence among practicing surgeons indicate that generous family policies alone do not remove structural barriers within surgical organizations [29, 30]. Population-level analyses link more generous paid-leave entitlements to better maternal mental health, underscoring the role of reliable childcare and income security in sustaining uninterrupted training [31, 32]. Consistent with international surveys documenting delayed childbearing, uneven parental-leave access, and caregiving-discouraging cultures, our results therefore point to program-level deficits rather than a lack of ambition among women [33–35].

### Towards structural change and gender equity

The ongoing underrepresentation of women in surgical leadership has often been attributed to the “glass ceiling”, which reflects deep-rooted gender norms, hidden biases, and a lack of female mentorship [36]. While verbal support for diversity has increased, meaningful institutional changes have remained limited.

Our findings emphasize actionable strategies, such as flexible work models, job-sharing leadership roles, on-site childcare, and transparent promotion pathways that account for family-related career interruptions. Studies have shown that part-time physicians - especially women - can be more efficient per hour than full-time staff, challenging assumptions about reduced output [37]. However, female respondents expressed a willingness to work longer hours if they had access to reliable adequate childcare, underscoring the important role of organizational infrastructure.

The surgical field urgently needs a paradigm shift: by integrating work-life balance as a core value, we can not only reduce turnover but also attract younger individuals to surgical careers [38]. This is not a question of ambition but of access. As Carnes et al. [26] emphasize, existing patriarchal structures continue to influence institutional culture, and breaking these down demands intentional reflection and long-term reform.

In summary, promoting gender equity in surgery requires a comprehensive strategy - visible female leadership, supportive institutional policies, and fair training models - to achieve both professional equality and the sustained health of the surgical workforce.

## Limitations

The studies’ cross-sectional design prevents causal inference, and the self-reported data may be subject to recall and social desirability bias. The overrepresentation of female respondents may reflect greater interest among those affected by the topic, potentially limiting the generalizability of gender comparisons. The inclusion of surgeons from different subspecialties may have introduced bias, as professional cultures and working conditions can vary across surgical disciplines. A further methodological limitation concerns construct asymmetry: both women and men were asked whether they would increase working hours if reliable childcare were available; however, the questionnaire lacked complementary items capturing men’s willingness to reduce working hours or assume additional caregiving. This framing risks perpetuating the stereotype that reconciliation is primarily a women’s issue. Future research should include gender-balanced, parallel items.

Additionally, findings are context-specific to the German healthcare system and may not be fully transferable to other countries with different training structures and family policies. Finally, while the survey instrument was based on a validated tool, the added items may affect internal consistency and comparability.

## Supplementary Information

Below is the link to the electronic supplementary material.

Supplementary Material 1

## Data Availability

The questionnaire is provided as Supplementary Material. De-identified data are available upon reasonable request.

## Abbreviations

CI: Confidence interval
NA: Not available
OR: Odds ratio
Ref.: Reference
