# Supplementary material for: Structural barriers to work-family reconciliation in surgery: a gendered analysis of career disruption and care responsibilities
Source: Updates Surg. 2026 Feb 6;78(2):487–97. doi: 10.1007/s13304-025-02489-3 (PMC13212764; doi:10.1007/s13304-025-02489-3)
Supplement: Supplementary file 1 — Supplementary Material 1 [file 13304_2025_2489_MOESM1_ESM.docx]

Appendix – Survey Questionnaire

Dear participant,

Thank you for taking the time to participate in our online survey. All responses are anonymous and the questionnaire will take approximately 30 minutes to complete.

This study aims to explore both structural and individual factors that contribute to a successful surgical career, with a particular focus on how gender may influence professional development. By completing the questionnaire, you consent to the use of your data for research and publication purposes.

You may skip any question if desired.

The questionnaire consists of the following five sections:
1. Education and Career History
2. Current Professional Status
3. Job Satisfaction
4. Future Career Perspectives
5. Personal and Family Background

If you have any questions, please contact v.nottberg@uke.de.

This survey includes a maximum of 68 questions.

**I) Education and Career History**

1. **What is your gender?**
    o Male
    o Female
    o Diverse
    o Prefer not to say
2. **Are you currently working at a university hospital?**
    o Yes
    o No
    o Prefer not to say
3. **Which surgical specialty have you completed or are you currently pursuing?** *(Multiple answers possible)*
    o General Surgery
    o Vascular Surgery
    o Cardiac Surgery
    o Trauma Surgery
    o Visceral Surgery
    o Thoracic Surgery
    o Plastic Surgery
4. **What was your average grade upon completing secondary school (Abitur)?**
5. **What was your final grade upon graduation from medical school?**
6. **Have you completed a doctoral degree (Dr. med.)?**
    o Yes
    o No
7. **How many years after graduation did you complete your doctoral degree?**
8. **What grade did you receive for your doctoral thesis?** *(Please enter a numeric value, e.g., 2.0)*
9. **How many years do/did you plan for your specialist training (residency)?**
10. **Has your specialist training taken longer than expected or is it foreseeable that it will take longer?**
     o Yes
     o No
11. **What were the reasons for prolonging your specialist training?** *(Multiple answers possible)*
     o One or more job changes during training
     o Part-time work during training
     o Parental leave during training
     o Lack of available rotation positions
     o Illness
     o Research activities
     o Stay abroad
     o Other (please specify)

**II) Current Professional Status**

1. **What is your current professional position?**
    o Resident
    o Specialist (Board-certified surgeon)
    o Senior physician / Consultant
    o Senior Consultant / Deputy chief physician
    o Head of department / Chief physician
    o Prefer not to say
2. **What is your current academic status at the university?**
    o Full Professor
    o Associate professorship
    o Assistant professor
    o Doctorate (Dr. med.) completed
    o Academic staff / research assistant
    o Prefer not to say
3. **Do you work full-time or part-time?**
    o Full-time
    o Part-time
4. **What is your average gross monthly income?**
    o < €3,000
    o €3,000–4,000
    o €4,000–5,000
    o €5,000–7,000
    o €7,000–8,500
    o €8,500–10,000
    o > €10,000
5. **What is your average gross monthly income including allowances?**
    o < €5,000
    o €5,000–7,000
    o €7,000–8,500
    o €8,500–10,000
    o > €10,000
6. **What is your regular weekly working time (excluding overtime)?**
7. **How many overtime hours do you work on average per week?**
    Paid overtime:
    Unpaid overtime:
8. **How often do you perform on-call or in-house emergency duties per month?**
    On-call duties (hours/month):
    In-house duties (hours/month):
9. **How much time (hours/week) do you spend on research during your free time?**
10. **How many scientific papers have you published (as first or last author)?**
11. **Do you use vacation days for research activities?**
     o Yes
     o No
     o Prefer not to say
12. **How many conferences have you attended in the past 12 months?**
13. **Have you acquired third-party research funding?**
     o Yes
     o No
     o Prefer not to say
14. **Approximately how much third-party funding have you been awarded in total?**
     (Please specify in €)
15. **How do you evaluate the following statements regarding research conditions?**
     o I agree
     o I disagree

- A scientific career is important to me.
- I am satisfied with the academic career opportunities.
- I would relocate to pursue a career opportunity (e.g., for a professorship).
- My family circumstances make relocation for career reasons impossible.
- I would like to engage (more) in research but lack the time.
- For me, research is also a personal hobby.
- I would conduct (more) research if the time/overtime required were compensated.
- A research sabbatical would increase my academic activity.
- In my case, research and family life are hardly compatible.
- I lack time to conduct research outside regular working hours.
- I lack the skills required for high-quality research.
- I consider the quality of my research to be good compared to other researchers in medicine.
- I have not been encouraged by supervisors to pursue research.

1. **Did you interrupt your surgical training for research purposes (e.g., research rotation, stay abroad)?**
    o Yes
    o No
    o Prefer not to say
2. **How long did you interrupt your surgical training for research purposes?**
    (Please specify in months)

### ****III) Personal and Family Background****

1. **What is your year of birth?**
    o 1950–1969
    o 1970–1979
    o 1980–1989
    o 1990–1999
    o After 2000
2. **What is your marital status?**
    o Married / Civil partnership
    o In a committed relationship
    o Single
    o Divorced
    o Widowed
    o Prefer not to say
3. **Is your partner currently employed?**
    o Yes
    o No
    o Prefer not to say
4. **What is the extent of your partner’s current employment?**
    o Full-time
    o Part-time
5. **Is your partner also working in the medical field?**
    o Yes
    o No
    o Prefer not to say
6. **Do you have a desire to have children?**
    o Yes
    o No
    o Undecided
    o Prefer not to say
7. **Have you given up the desire to have children for professional reasons?**
    o Yes
    o No
    o Prefer not to say
8. **Have you postponed having children for professional reasons?**
    o Yes
    o No
    o Prefer not to say
9. **Do you have children?**
    o Yes
    o No
    o Prefer not to say
10. **How many children do you have?**
11. **At what stage in your career did you have your (first) child?**
     o Before medical school
     o During medical school
     o During specialist training
     o After specialist training (no senior position)
     o After specialist training with senior position
     o After specialist training with chief position
12. **How are/were your child(ren) primarily cared for during the day?**
     o By myself
     o By my partner
     o By other family members
     o By a nanny, au pair, friends, or acquaintances
     o By a public institution
     o Other (please specify)
     o Prefer not to say
13. **Who is responsible for organizing your child(ren)’s activities (e.g., appointments, parent-teacher meetings, transport to hobbies/friends)?**
     o Myself
     o My partner
     o Relatives / Friends
     o Childcare provider (e.g., au pair, babysitter)
     o Other (please specify)
14. **Would you like to work more if reliable childcare were available?**
     o Yes
     o No
     o Prefer not to say
15. **Have you or your partner ever interrupted your career due to childcare responsibilities?**
     o Yes, I interrupted my career
     o Yes, my (spouse/partner) interrupted their career
     o Yes, my partner has suspended their career indefinitely for childcare
     o No, neither of us has interrupted our careers
     o Other
16. **How long was/were the career interruption(s)?**
     Own interruption (in months):
     Partner’s interruption (in months):

### ****IV) Parental Impact and Gender-Related Experiences****

1. **In case of a child’s illness, who provides care?**
    o I do
    o My partner does
    o We alternate
    o Prefer not to say
    o Other (please specify)
2. **Have you experienced reassignment to non-surgical tasks due to your pregnancy or your partner’s pregnancy?**
    o Yes
    o No
    o Partially
    o Prefer not to say
3. **Do you feel your opportunities for advancement have been limited due to having children?**
    o Yes
    o No
    o Partially
    o Prefer not to say
4. **How do you assess the following statements?**
    o Strongly agree
    o Somewhat agree
    o Somewhat disagree
    o Strongly disagree

- Surgical training suffers from career breaks due to parenthood (e.g., parental leave, maternity leave)
- Surgical training suffers from breaks due to research activities (e.g., research stay abroad)
- Male physicians with children receive less adequate surgical training
- Female physicians with children receive less adequate surgical training

1. **How many hours per week do you spend on household work and/or childcare?**
2. **How many hours per week does your partner spend on household work and/or childcare?**
3. **Do you believe there are gender-based differences in how leadership skills are evaluated?**
    o Yes
    o No
    o Unsure

### ****Job Satisfaction****

1. **What were your main expectations regarding your current position?**
   (Response options: Very important – Important – Less important – Not important)

- High-quality clinical training
- Opportunity to conduct research
- Structured training / compliance with training timelines
- Career advancement / promotion opportunities
- Support for professional development
- Good salary
- Enjoyment at work
- Positive working environment
- Family-friendliness
- Autonomy in work organization
- Predictable working hours and job security
- Recognition

1. **To what extent have these expectations been fulfilled?**
   (Response options: Fully met – Mostly met – Hardly met – Not met at all)

- High-quality clinical training
- Opportunity to conduct research
- Structured training / compliance with training timelines
- Career advancement / promotion opportunities
- Support for professional development
- Good salary
- Enjoyment at work
- Positive working environment
- Family-friendliness
- Autonomy in work organization
- Predictable working hours and job security
- Recognition

1. **Please answer the following questions regarding your physical and mental well-being:**
   (Response options: Never – Rarely – Sometimes – Often – Always)

- Do you feel physically exhausted?
- Do you feel emotionally exhausted?
- Do you go to work even when feeling ill?
- Do you feel that your work is meaningful?
- Do you have trouble unwinding after work?
- Do you feel stressed by difficult decisions in patient care?

1. **Personality traits – to what extent do the following statements apply to you?**
   (Response scale: Does not apply at all – Mostly does not apply – Rather does not apply – Neither agree nor disagree – Rather applies – Mostly applies – Fully applies)

- Extroverted, enthusiastic
- Critical, argumentative
- Reliable, self-disciplined
- Anxious, easily upset
- Open to new experiences, imaginative
- Reserved, quiet
- Compassionate, warm
- Disorganized, careless
- Calm, emotionally stable
- Conventional, uncreative

1. **To what extent do you feel supported in your professional activities by the following groups?**
   (Response options: No support – Moderate support – Strong support – Full support – Not applicable)

- Supervisors
- Colleagues
- Staff members
- Spouse / Partner
- Family
- Friends and acquaintances

1. **How satisfied are/were you with the following aspects of your clinical training and research opportunities?**
   (Response options: Very satisfied – Rather satisfied – Rather unsatisfied – Very unsatisfied – Not applicable)

- Career development discussions with supervisors
- Opportunities for clinical training
- Research leave / protected research time
- Opportunities to attend conferences
- Inclusion in research projects
- Opportunities to develop own projects and apply for grants
- Availability of supervision
- Support in dealing with critical incidents

1. **Have you personally experienced gender-based discrimination or prejudice during your career?** (Multiple answers possible)

- Yes, during promotion
- Yes, during salary negotiations
- Yes, in the daily work environment
- Yes, in the form of sexual harassment
- No
- Prefer not to say

1. **Have you had a mentor during your career?**
    o Yes
    o No
    o Prefer not to say
2. **What was your mentor’s gender?**
    o Male
    o Female
    o Diverse
    o Prefer not to say
3. **Do you believe your gender has had an influence on your professional career path so far?**
    o Yes
    o No
    o Prefer not to say

### ****V) Career Goals****

1. **Would you say you have career ambitions?**
    o Yes
    o No
    o Unsure
    o Prefer not to say
2. **What is your career goal?** (Multiple answers possible)

- Leadership position (Chief) at a university hospital
- Leadership position (Senior) at a university hospital
- Leadership position (Chief) at a non-university hospital
- Leadership position (Senior) at a non-university hospital
- Senior physician at a university hospital
- Senior physician at a non-university hospital
- Specialist at a university hospital
- Specialist at a non-university hospital
- Career outside of clinical care (e.g., in industry)
- Physician in outpatient care
- Physician in academic research
- Other (please specify)

1. **Have you completed or are you planning to complete a postdoctoral habilitation?**
    o Yes, already completed
    o Yes, planning to complete
    o No
    o Prefer not to say
2. **Why are you not pursuing (or no longer pursuing) a habilitation?** (Multiple answers possible)

- Too much effort for too little return
- Lack of interest
- Not relevant to my career goals
- Incompatibility with family responsibilities
- Lack of leave/funding opportunities
- Other (please specify)

1. **How many years after your doctoral degree did you complete your habilitation?**
2. **What were or are your reasons for pursuing a habilitation?** (Multiple answers possible)

- To qualify for a professorship
- To qualify for a chief physician position
- Interest in research and science
- To improve promotion prospects outside academia
- Interest in teaching
- To expand personal development opportunities
- To gain greater independence
- Other (please specify)

1. **How helpful do you consider the following measures to enhance the appeal of leadership roles in academic medicine?**
   (Response options: Extremely helpful – Very helpful – Moderately helpful – Slightly helpful – Not helpful at all)

- Flatter hierarchies
- Improved financial resources
- Options for leadership job-sharing (“top sharing”)
- Dual-career support for partners
- Gender balance at leadership levels
- Measures to increase the societal value of research and teaching
- More flexible working hours (e.g., time accounts)
- Programs to improve work-family balance
